# 732 Economic and Environmental Benefits of Telemedicine Expansion

**DOI:** 10.1093/jbcr/irad045.207

**Published:** 2023-05-15

**Authors:** Sam Miotke, Emily Oxton

**Affiliations:** Regions Hospital, St. Paul, Minnesota; Regions Hospital, St. Paul, Minnesota; Regions Hospital, St. Paul, Minnesota; Regions Hospital, St. Paul, Minnesota

## Abstract

**Introduction:**

The COVID-19 pandemic mandated a robust expansion of our program’s telemedicine bandwidth. Despite the lifting of pandemic restrictions, the number of telemedicine visits has stayed consistent, which is a credit to both the quality of care provided and to the convenience of receiving care via computer or smartphone. We sought to quantify some aspects of this convenience to more thoroughly understand the benefits of our program's expansion.

**Methods:**

We examined home addresses for all patients seen via telemedicine from June, 2021 through May, 2022. We estimated the distance that telemedicine saved each patient by searching a navigation website for the most time efficient route from their hometown to our burn center. Patients who lived in the same city as our center were assigned a distance of 1 mile. Hours saved were estimated by assuming a driving speed of 70 miles per hour. Fuel money saved was estimated by assuming an average fuel economy of 24.2 miles per gallon and an average gas price of $3.61. The overall carbon footprint was estimated by assuming an average CO2 emission of 411 grams/mile.

**Results:**

1,794 patient visits were conducted via telemedicine over this 12 month span. The total estimated amount of roundtrip travel saved was 621,718 miles which equated to 8,882 hours and $92,744 dollars saved. The carbon footprint saved was 255.5 metric tonnes. On average, our telemedicine program saved an estimated 4.95 hours and $51.70 in gasoline per patient-visit.

**Conclusions:**

Even when ignoring the potential hurdles of transportation access, family care, missed time at work, and parking fees there are clear quantifiable reasons behind the program’s popularity. The environmental benefit is an important byproduct of its convenience.

**Applicability of Research to Practice:**

Our telemedicine program requires dedicated staffing and significant patience with technology but has remained popular due to the outlined advantages and has facilitated the expansion of our program into a broader catchment area.

